# Regulatory T lymphocytes as a treatment method for rheumatoid arthritis – Superiority of allogeneic to autologous cells

**DOI:** 10.1016/j.heliyon.2024.e36512

**Published:** 2024-08-30

**Authors:** Joanna Chmiel, Mariusz Stasiak, Maria Skrzypkowska, Lucjan Samson, Piotr Łuczkiewicz, Piotr Trzonkowski

**Affiliations:** a University Clinical Centre in Gdańsk, Second Clinic of Orthopaedics and Kinetic Organ Traumatology, Poland; b Faculty of Medicine, Medical University of Gdańsk, Poland; c Department of Medical Immunology, Faculty of Medicine, Medical University of Gdańsk, Poland

**Keywords:** Regulatory T cells (tregs), Rheumatoid arthritis (RA), Adoptive cell therapy (ACT)

## Abstract

Cellular therapies utilizing regulatory T cells (Tregs) have flourished in the autoimmunity space as a new pillar of medicine. These cells have shown a great promise in the treatment of such devastating conditions as type 1 diabetes mellitus (T1DM), systemic lupus erythematosus (SLE) and graft versus host disease (GVHD). Novel treatment protocols, which utilize Tregs-mediated suppressive mechanisms, are based on the two main strategies: administration of immunomodulatory factors affecting Tregs or adoptive cell transfer (ACT). ACT involves extraction, *in vitro* expansion and subsequent administration of Tregs that could be either of autologous or allogeneic origin. Rheumatoid arthritis (RA) is another autoimmune candidate where this treatment approach is being considered. RA remains an especially challenging adversary since it is one of the most frequent and debilitating conditions among all autoaggressive disorders. Noteworthy, Tregs circulating in RA patients' blood have been proven defective and unable to suppress inflammation and joint destruction. With this knowledge, adoptive transfer of compromised autologous Tregs in the fledgling clinical trials involving RA patients should be reconsidered. In this article we hypothesize that incorporation of healthy donor allogeneic Tregs may provide more lucid and beneficial results.

## Highlights

• Adoptive transfer (ACT) of autologous Tregs helps to treat autoimmune diseases.
• Tregs become defective in the inflammatory conditions.
• ACT of allogeneic Tregs may become more beneficial than transfer of autologous cells.
• Creating an allogeneic immune cell bank system could offer advantages.

## Abbreviations

RA -: Rheumatoid Arthritis
QUEST-RA -: Quantitative Patient Questionnaires in Standard Monitoring of Patients with Rheumatoid Arthritis
DMARDs –: Disease-Modifying Antirheumatic Drugs
JAKi –: Janus Kinase inhibitor
Tregs –: Regulatory T lymphocytes
T1DM -: Type 1 Diabetes Mellitus
SLE –: Systemic Lupus Erythematosus
GVHD –: Graft versus Host Disease
ACT –: Adoptive Cell Transfer/Therapy
CD –: Cluster of Differentiation
IL-2 –: Interleukin 2
Foxp3 –: Forkhead box P3
tTregs –: Thymus-derived Regulatory T lymphocytes
pTregs –: Peripherally-derived Regulatory T lymphocytes
iTregs –: Induced Regulatory T lymphocytes
TGF-β –: Transforming Growth Factor beta
ACP -: Antigen Presenting Cell
TNF-α –: Tumour Necrosis Factor alpha
PB –: Peripheral Blood
SF –: Synovial Fluid
OA –: Osteoarthritis
Ab –: Antibody
IFN-γ –: Interferon gamma
Tconv -: Conventional T cells
MSC –: Mesenchymal Stem Cell
atRa -: all-*trans* Retinoic Acid
TCR –: T cell Receptor
MHC –: Major Histocompatibility Complex
CAR –: Chimeric Antigen Receptor
CRISPR -: Clustered Regularly Interspaced Short Palindromic Repeats
DAS28 -: Disease Activity Score
CDAI -: Clinical Disease Activity Index
ACR -: American College of Rheumatology
EULAR -: European League Against Rheumatism
Teffs –: T effector cells
QOL –: Quality of Life
HLA –: Human Leukocyte Antigen
UCB –: Umbilical Cord Blood
ARDS –: Acute Respiratory Distress Syndrome
GMP –: Good Manufacturing Practice
HSCT –: Hematopoietic Stem Cell Transplantation

## 1 Introduction

Rheumatoid arthritis (RA) is a systemic autoimmune disease characterized by chronic synovial inflammation, leading to cartilage damage, bone erosion and, ultimately, severe joint destruction. Autoreactivity and dysregulation of self-tolerance are thought to be responsible for the disease onset. RA is one of the main causes of permanent work disability, associated with many social consequences [1]. Between January 2005 and October 2006, the QUEST-RA (Quantitative Patient Questionnaires in Standard Monitoring of Patients with Rheumatoid Arthritis) project was conducted to achieve quantitative clinical assessment of patients with rheumatoid arthritis seen in standard rheumatology care in 15 countries. With 4363 included participants, this international multi-centre cross-sectional database provided a thorough overview of clinical status and treatments of patients with RA. Data on demographic, disease and treatment-related variables were collected and analyzed using descriptive statistics, enriching our knowledge on RA specific demographic characteristics. The QUEST-RA database has continued to grow and in June 2009 included over 8000 patients from 86 clinics in 32 countries. Based on collected data, the statistcal analysis was performed, showing that work disability rates remain high among people with RA during this millennium. More than one third (37 %) of participants reported subsequent work disability because of RA [2].

Current therapies: conventional synthetic DMARDs, targeted synthetic DMARDs (JAK inhibitors) and biological DMARDs can induce remission in approximately 70 % of the patients, however, remaining 30 % experience incurable and extremely debilitating affliction [3,4]. Nowadays, all efforts are given to identify new therapeutic approaches, especially those focused on immune homeostasis [5,6].

Regulatory T lymphocytes (Tregs) are a vital part of the adaptive immune system, responsible for preventing excessive inflammatory responses and maintaining immune balance. They are a specialized subpopulation of T helper cells, essential for inhibiting pathological reactions towards self-antigen. Given their unique ability to control aberrant autoreactive responses, there has been a significant interest in targeting Tregs therapeutically [7]. The pivotal role of Tregs in the maintenance of immune balance gave rise to a number of studies on their physiology and therapeutic potential [8]. Currently, regulatory T cell-based therapies are way past the *in vitro* phase, demonstrated in murine models. They've entered the clinical phase and some promising results have been reported repeatedly. Over the years, a lot of data has been collected, showing a great efficacy of Treg-based treatment approaches, not only in autoimmune diseases but also in GVHD – one of the biggest challenges to overcome in bone marrow transplantation [9,10].

As for RA, the size of its clinical data repository remains limited when compared to T1DM or GVHD, which are the diseases that have drawn researchers' attention from the very beginning of the Treg-based therapies era. Nevertheless, in several completed trials, Tregs have been proven useful in the management of RA, helping to overcome impaired functions and reduced frequencies of factors responsible for maintaining immune balance. The most promising Treg-based treatment protocol seems to be the one involving adoptive transfer of *in vitro* expanded Tregs [11]. Adoptive T cell therapy (ACT) used in autoimmunity usually utilizes polyclonal autologous lymphocytes, extracted and isolated from the peripheral blood of the treated subject. Although peripheral blood of healthy individual usually provides a sufficient amount of Tregs, they present a terminally differentiated memory phenotype, which reduces not only the expansion capacity, but also the likelihood of successful engraftment and survival [12]. This situation changes in autoimmune conditions, such as RA, in which we observe Tregs reduced frequencies and impaired functions. Moreover, different reports have shown that high plasticity of Tregs makes them particularly susceptible to inflammatory signals [13]. As the result, their regulatory and anti-inflammatory profile becomes defective. This causes inability to control cytokine-activated T cell functions and results in autoimmune-mediated damage to the healthy tissue. While Tregs protect body against autoaggression in healthy individuals, under autoimmune conditions their immunosuppressive properties become insufficient and even contribute to exacerbation of the disease [14,15]. This unfortunate phenomenon seems to be one of the culprit behind the inability to achieve a full therapeutic success of polyclonal autologous ACT [16,17].

We believe that changing the source of regulatory lymphocytes to a healthy donor could bring more certain and valuable conlusions and better understanding of the complex mechanisms behind the immune tolarance in RA. In this narrative review, we provide a basic background on the regulatory T lymphocytes and their role in the treatment of autoimmunity. We also hypothesize about a novel approach in the treatment of RA, based on adoptive transfer of allogeneic regulatory T cells.

## 2 The aim of this narrative review

Our aim was to give a brief overview of studies focused on harnessing regulatory T lymphocytes as a new therapeutic approach to treat autoimmune and other immunological diseases, especially RA. In this review, we highlight both the advances and the challenges in this arena, such as Tregs declined stability under inflammatory conditions. We also summarize available data (as of June 2024) on the clinical trials involving *in vivo* Tregs enhancement in RA patients, as well as the first implementation of allogeneic Tregs in the treatment of different diseases.

## 3 Methods

A literature search was performed in October and November 2023 and updated in January 2024 in PubMed database. Additional abbreviated update was performed in June 2024. The database was searched using a combination of subject headings and free‐text terms in the title and abstract fields. Highlighted keywords for searching were: regulatory T cells, adoptive cell transfer, rheumatoid arthritis. We focused our interest on most recent papers (date of publication 2014 to present), especially extensive meta-analyses summarizing the data from the last two decades.

As for for the clinical trials research, database used was ClinicalTrials.gov. The date of our investigation was June 2024, with applied search criteria: Tregs; regulatory T lymphocytes; rheumatoid arthritis; allogeneic Tregs.

For practical purposes, the search strategies were limited to English language as no resources were available to translate papers in other languages.

## 4 Regulatory T lymphocytes

In 1995 Sakaguchi et al. reported a subset of T-helper lymphocytes (CD4^+^) that maintain immune homeostasis by suppressing proliferation of effector T-cells [18]. Those lymphocytes expressed characteristic receptor for IL-2 (CD25). Over the years, more data about Tregs properties has been collected, suggesting the important role of Foxp3 transcription factor, which defines Treg identity and function. The population of CD4(+)CD25(+)Foxp3(+)CD127(low/-) T lymphocytes is currently recognized as the major immunoregulatory subset since it has been the most thoroughly studied [19]. These cells are often called naturally-occurring Tregs (nTregs) and consist of two subgroups: thymus-derived Tregs (tTregs), produced by the thymus as a functionally distinct and mature population, and peripherally-derived Tregs (pTregs), generated extrathymically at peripheral sites under certain conditions.

Tregs differentiation from naïve CD4(+) cells is regulated by TCR signaling that commit precursors to a regulatory lineage. Tregs can also be induced from non-Tregs *in vitro*, e.g. by antigenic stimulation in the presence of TGF-β and IL-2 (induced Tregs (iTregs)) [20]. Interestingly, there is an increasing evidence that some CD8(+) T cells and B cells also show suppressive activity and have major impact on the immune homeostasis [21,22]. These findings confirm extremely complex nature of specific interactions between every cell in human immune system.

**Fig. 1:**
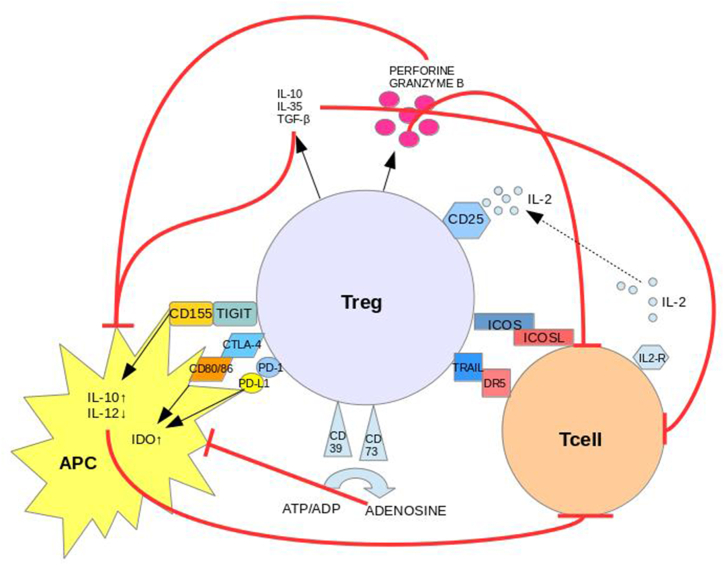
Mechanisms of Treg suppression. CTLA-4 binds to CD80/CD86 on APC (Antigen Presenting Cell), inhibits antigen presentation and increases IDO (Indoleamine 2,3-dioxygenase) expression. IDO pathway induces T cells anergy and enhances Tregs function. PD-1 (Programmed Death Receptor 1) binds to its ligand on APC resulting in suppression of antigen-reactive activity of Teff (effector T lymphocyte). TIGIT (T cell Immunoreceptor with Ig and ITIM domains) presented on Treg interacts with CD155 on dendritic cell and enhances the expression of IL-10, thus specifically suppresses proinflammatory Th1 and Th17 cells. Anti-inflammatory cytokines are also directly produced by Tregs. Exosomes (extracellular vesicles) secreted by Tregs contain many specific biological molecules, which are delivered to target cells and modulate immune responses by inhibiting T cell proliferation, inducing apoptosis and changing the cytokine expression profiles of target cells. Activated Tregs cells also utilize granzyme B and perforin to disrupt protective barrier of cell membrane and destroy integrity of the target cell. CD39/CD73 expressed on Treg degrade ATP into extracellular adenosine, which is implicated in the immunoregulatory activity by inhibiting antigen presentation by APCs. TRAIL/DR5 (TNF-Related Apoptosis-Inducing Ligand/Death Recptor 5) interaction activates caspase-8 to induce Teff apoptosis. ICOS (Inducible T-cell Co-Stimulator) signaling can mediate Tregs survival by fine-tuning the expression of multiple anti-apoptotic molecules and anti-inflammatory cytokines. CD25, also konown as IL-2 receptor (IL-2R), is expressed at high levels on the surface of Tregs. IL-2, though not produced by Tregs, is essential for their development. Tregs compete for IL-2 secreted by effector cells and prevent them from acquiring the sufficient amount for proliferation and activation.

Naturally-occurring Treg lymphocytes present their regulatory effect via cell-cell contact or by producing anti-inflammatory cytokines [14] [Fig. 1]. As mentioned before, there have been some discoveries suggesting that under certain conditions, such as inflammatory environment, Tregs start to present pro-inflammatory features and fail in preventing autoaggression [23]. In 2014, a ground-breaking paper by Jeffrey Bluestone's group showed, using mice fate mapping analysis, that Tregs can start to produce IL-17 due to the down-regulation of Foxp3 gene transcriptional activity, triggered by autoimmune arthritis environment [24]. These IL-17 producing cells are particularly arthritogenic and capable of inducing a severe chondral damage. Tumour necrosis factor α (TNF-α), present in inflammed joints in high concentrations, is one of the cytokines that are responsible for the impaired Treg cells function [15,Table 1].

**Table 1:** Impairment of Tregs suppressive functions in inflammatory conditions.

Group of factors responsible for impaired Tregs suppressive function	Main representatives	Effect on Tregs	Reference
Cytokines	IL-6 IL-1 IL-21 TNF-α*	Tregs proliferation ↓ Tregs apoptosis ↑ Teff resistance to suppression ↑ Foxp3 expression ↓	[[25], [26], [27], [28], [29]]
Transcription factors&Signaling pathways	NF-κB HIF-1 IRFs AP-1 JAK/STAT PI3K TLR Hhex Foxp1 Eos YAP Notch	Tregs proliferation ↓ Tregs stability ↓ Foxp3 expression ↓ CD-25,CTLA-4 expression ↓ Foxp3 modification ↑	[[30], [31], [32], [33], [34]]
Post-translational modification	Ubiquitination phosphorylation O-GlcNAcylation acetylation methylation	Foxp3 expression ↓ Foxp3 stability ↓ Dysregulation of Th17/Treg balance ↑	[25,[35], [36], [37]]
miRNAs	miR-146a miR-301a-3p miR-142-3p miR-23a-3p miR-181a/b miR-568 miR-17	Signaling pathways impairment ↑ Dysregulation of Th17/Treg balance ↑ Tregs apoptosis ↑ Tregs stability ↓ CTLA-4 expression ↓ Foxp3 expression ↓ Foxp3 modification ↑	[25,[38], [39], [40]]
Proteins	PAR Mst1/Mst2 mTOR	Signaling pathways impairment ↑ Foxp3 modification↑ Foxp3 expression ↓	[33,41,42]
Cells	Th17 Th1 Nks	Tregs apoptosis ↑ Tregs proliferation ↓ Foxp3 expression ↓	[25,27,43,44]

Abbrevations: IL – Interleukin; TNF-α – Tumor Necrosis Factor alpha; NF-κB - Nuclear Factor *kappa*-light-chain-enhancer of activated B cells; HIF-1 - Hypoxia-inducible Factor 1; IRFs - Interferon Regulatory Factors; AP-1 - Activator Protein-1; JAK/STAT - Janus Kinase/Signal Transduction and Transcription Activation; PI3K – Phosphatidylinositol 3-Kinase; TLR - Toll-like Receptors; Hhex - Homeobox protein Hhex; Foxp - Forkhead box P; Eos - transcription factor of the Ikaros family; YAP - Yes-associated Protein; PAR - Protease-activated Receptor; Mst1/Mst2 – Hippo kinases; mTOR - mammalian Target of Rapamycin kinase; Th – T helper lymphocytes; NKs – Natural Killer cells; CTLA-4 - Cytotoxic T lymphocyte–associated Protein 4.

* TNF-α has contradictory effects on the functions of Tregs [25].

### 4.1 Tregs in autoimmune diseases

Treg-mediated immunosuppression provides self-tolerance and any failure in that system leads to some kind of autoimmune disorder [45]. The pathological changes we observe as the result of immune imbalance may affect multiple systems and organs of the human body. Autoimmune diseases present a wide variation of symptoms: they may become chronic or acute; may present episodes of remission followed by aggressive relapses; the course of the disease may vary from mild to severe. Accordingly, their response to wide range of available treatment methods depends on many different factors and mechanisms, often not fully understood. Currently there are more than 80 types of autoimmune diseases described in the literature. They affect approximately 10 % of the global population [46]. Examples of such diseases include type 1 diabetes mellitus, multiple sclerosis, psoriasis and rheumatoid arthritis.

#### 4.1.1 Tregs in RA

The prevalence of RA varies globally, with generally higher occurrence in the industrialized countries [47]. In the last years, RA was reported to be the most disabling disease, causing early departure from professional career path and enormous limitations in the social life area [2]. Immune regulation in RA is extremely complex and may be achieved through a direct cell-cell contact or paracrine signalling. Tregs mediate the downrgulation of antigen-presenting cells (APC) and also regulate the metabolism in effector T cells [8,48] [Fig. 1]. In the last two decades, there has been an explosion in the research describing the role of Tregs and their relevance in different autoimmune disorders, enriching our knowledge about the etiology and pathogenesis of autoimmunity.

It has been shown that Tregs are frequently found subpopulation in the peripheral blood (PB) and synovial fluid (SF) of RA patients - with significantly higher numbers in the latter [49]. In 2016, Morita et al. presented a meta-analysis of 31 studies, confirming that the proportion of Tregs, defined by both Foxp3 and CD25, was higher in SF than that in PB among RA patients [50]. However, enhanced presence of regulatory cells is clearly not sufficient to prevent autoimmunity. It is believed that suppressive function of Tregs is impaired partly because of pro-inflammatory cytokines (e.g.TNF-**α**) produced by synovium [51,52]. Unparalleled plasticity of Tregs is responsible for the loss of their anti-inflammatory phenotype in highly inflammatory environment of RA [53,54] [Table 1]. As compared to healthy individuals, the quantity of Tregs in the synovial fluid of RA patients may be higher, but this increased number does not compensate for functional impairment. Tregs affected by RA simply fail to suppress auto-reactive effector lymphocytes and start to contribute to the progression of the disease via number of mechanisms, such as Treg conversion to Th17 (Bluestone et al.) [24,55,56].

### 4.2 Tregs in non-autoimmune diseases

Tregs function is not limited to self-tolerance and autoimmunity. Over the years, several additional properties have been suggested [57]. Interestingly, emerging evidence suggest that regulatory T lymphocytes intervene in the inflammatory process that drive osteoarthritis (OA), considered for decades a simple degenerative disease. Some groups demonstrated that decreased Tregs responses may contribute in the pathogenesis of chondral damage in OA [58,59].

The role of Tregs have been also described in the regenerative process after stroke or myocardial infarction [[60], [61], [62]]. If so, it may imply similar regenerative activity around the joints.

Furthermore, it appears that the adaptive immunity during severe SARS-CoV-2 infection is dysregulated and the number of Tregs significantly decreased. At the same time, their functional suppressive potential seems to be elevated with high expression of molecular markers [63].

The undeniable role of regulatory T cells in the pathophysiology of cancer should also not be forgotten. In tumor-bearing individuals, Tregs compromise the response of immune system against tumour, and therefore accelerate the progression of cancer [[64], [65], [66]].

## 5 Treg-based therapies

To date, two main strategies have been developed in the field of Treg-based therapies: the administration of immunomodulatory factors enhancing the number or function of Tregs and ACT - adoptive transfer of *in vitro* expanded Treg lymphocytes. The first one rely on *in vivo* protocols, involving specific immulomodulatory agents targeting the key molecules of Tregs maintenance, thus improving Treg-mediated immune tolerance [67]. These therapies involve treatment with cytokines such as low-dose IL-2, IL-2/Anti-IL-2 Ab complex and IL-4, IL-12, IFN-γ– in murine models [10,48]. However, the most promising results were provided by clinical trials focused on adoptive immunotherapy [68].

ACT refers to the *ex vivo* manipulation of T cells and their subsequent reinfusion into patient. While numerous in-human trials have demonstrated that Tregs reduce the severity of autoimmune reactions, several impediments still remain, including Tregs variability and the practical need for their effective *in vitro* expansion techniques.

Many studies on the heterogeneity of regulatory T lymphocytes population revealed distinct subsets with different functions in the control of the immune homeostasis and induction of peripheral tolerance. All these Tregs subsets and their specific properties can be exploited to develop novel treatment methods [69].

As mentioned before, the number of Tregs circulating in the peripheral blood is significantly lower than that of other lymphocytes, making them challenging to extract and multiply. There have been also some controversies on the proper identification method for Tregs, which caused a lack of clarity in the firstly emerged studies [50]. Fortunately, consensus on coherent identity markers has been achieved, followed by introduction of specific protocols that allow *in vitro* expansion of Tregs in sufficient amount [[70], [71], [72]].

Efficient clinical-scale Treg product manufacturing involves three key steps – isolation, adequate *ex vivo* expansion, while maintaining regulatory properties, and quality assessment [73]. Currently, the CD4(+)CD25(+)Foxp3(+)CD127(low/-) phenotype is most commonly isolated from Tregs population through flow cytometry or immunobead-separation methods. Tregs can be further distinguished from resting conventional T cells (Tconv) by hypomethylation of Foxp3, which enables to recognize regulatory profile at the genetic level. Tregs are then activated in the presence of high levels of exogenous interleukin-2 (IL-2). Next step is cell proliferation stimulated by anti-CD3 and anti-CD28 (aCD3/28) beads [74].

Various approaches have been explored to enhance the proliferation, function and stability of Tregs, including modifying culture conditions or adding drugs, e.g. Treg + MSC (Mesenchymal Stem Cells) co-culture, rapamycin, IL-6 and TNF-α [75,76]. Given the low frequency of Tregs in human peripheral blood, a feasible approach is the *in vitro* generation of potent Tregs (iTregs) from non-Tregs, since they are much higher in numbers and relatively easy to isolate**.** iTreg cells can develop from naïve CD4(+)Foxp3(−)T cells upon TGF-β stimulation. However, unlike tTregs, iTregs are highly unstable, which is a significant obstacle to their use for adoptive immunotherapy [77]. Interestingly, all-*trans* retinoic acid (atRA), a vitamin A metabolite, has been proven essential in regulation of a wide range of biological processes, including the *in vitro* enhancement of Tregs function and stability. This development has become especially useful in the manufacturing of both nTregs and iTregs for adoptive transfer purposeses [78].

Most Tregs manufacturing methods have been developed and validated based on autologous polyclonal Treg products. Moreover, most clinical trials have also utilized only polyclonal Treg cells populations, acquired from the treated subject and subsequently expanded *in vitro*. However, evidence from pre-clinical studies has clearly shown that antigen-specific Treg-based ACT is vastly superior to polyclonal cell-based [79]. The most basic method to obtain antigen-specific product includes antigen-stimulated Tregs expansion. This approach is mainly used in transplantation, to prevent graft rejection, and involves the use of APCs (Antigen Presenting Cells) from the graft donor to specifically stimulate alloreactive Tregs from the recipient. However, the implementation of this method in autoimmunity is limited due to the lack of knowledge of patient-specific TCRs (T cell receptors), which recognize disease-relevant MHC (Major Histocompatibility Complex)-peptide complexes [9].

Accessibility and safety of polyclonal Tregs have made them more frequently used in ongoing and past clinical trials. Polyclonal Tregs used in ACT may be autologous, derived from peripheral blood of the treated subject (the most popular source), or allogeneic – isolated from a healthy donor. Umbilical cord blood (UCB) could also become the source of allogeneic regulatory cells [80].

### 5.1 Genetic engineering of Tregs

The emergence of advanced gene editing techniques has opened the door to new methods of Tregs modifications, creating therapeutics with improved specificity and function. These strategies include advanced generation of highly antigen-specific Tregs, based on viral transduction with CAR (Chimeric Antigen Receptor)- or engineered TCR-encoding vector, as well as genome editing, utilizing CRISPR technology [9,81,82]. Furthermore, transgenic overexpression of Foxp3 in antigen-specific conventional CD4(+) lymphocytes has become an appealing way to produce a larger source of suppressive cells for infusion, thus overcoming the challenges of low precursor frequency [83].

Without a doubt, technological advance in genomics and cell manufacturing has made such individualized therapies possible, unfortunately, they are still extremely expensive, thus difficult to incorporate on a larger scale. Furthermore, there are several risks associated with Tregs genetic engineering, such as off-target disruption of essential genes or carcinogenesis associated with viral transduction and gene editing. There is a pressing need for careful monitoring of ongoing clinical trials utilizing genetically engineered Tregs, to further characterize the long-term risks and ensure safety of such therapies [84,85].

### 5.2 Treg-based therapies in RA

Currently, synthetic conventional, synthetic targeted and biological DMARDs are widely used in clinical practice and allow to achieve remission in approximately two out of three patients [3,86,87]. In 2018 Chinese cross-sectional observational study, including a total of almost 2000 RA participants, showed that the proportions of patients who fulfilled remission criteria was only 1.75 %–10.90 % (depending on the method used to evaluate response to the treatment – DAS28, CDAI, ACR/EULAR). The results indicated that the rate of disease activity remission achieved by currently accepted treatment regimen is disappointing. Moreover, adverse effects of applied drugs occurred in approximately one in five patients [88]. New approach is needed for those who do not respond well to standard disease-modifying drugs. To meet expectations, researchers started to develop several new therapies, especially based on the autoimmune background of RA and Tregs unique properties.

First strategy – application of cytokines or specific antibodies that target Tregs that circulate in the body of the treated patient, provoking the increase in Tregs frequency or suppressive function – has been repeatedly proven effective. These studies have been successfully introduced into human trials more than 10 years ago and presented highly promising results in many autoimmune diseases, such as SLE, Sjögren syndrome, dermatomyositis [[89], [90], [91]]. Unfortunately, in RA population these cytokines have been used only very recently [Table 2]. One of the most interesting study was TRANSREG initiative that compared the biological and clinical responses to the administration of low doses IL-2 across 14 autoimmune and inflammatory pathologies over the course of 7 years (2014–2021). Primary endpoint assessed during TRANSREG study was the change in Tregs frequency, expressed as a percentage of total CD(+) count, after the induction period, compared with baseline. Secondary biological endpoints were changes in Tregs and other immune cells (e.g. CD3(+) T cells, CD4(+) T cells, CD8(+) T cells, CD19(+) B cells, NK cells/mm3) at specific periods of time, compared with baseline, and and ratio Tregs/Teffs in induction and maintenance period.

**Table 2:** A brief summary of different approaches to *in vivo* Tregs enhancement in clinical trials involving RA patients. Database used for the research: ClinicalTrials.gov. Search criteria: Tregs; regulatory T lymphocytes; rheumatoid arthritis.

Therapeutic	Results	**ClinicalTrials.gov ID**	Reference
Low-dose IL-2	Treg ↑	NCT01988506*	[93,94]
	CGI ↓		
Abatacept	Treg ↑	NCT02885818	[95]
	Breg ↑		
	IL-10 ↑		
	TGF-β ↑		
	Th1 ↓		
	Th17 ↓		
	VAS ↓		
	CRP ↓		
Low-dose hrIL-2 (+MTX +Loxoprofen)	Tregs ↑	NCT02467504	[96]
	Th17 ↓		
	ACR20 ↓		
	CDAI ↓		
	SDAI ↓		
Autologous BM-MSCs	Treg ↑	NCT03333681	[[97], [98], [99]]
	TGF-β ↑		
	Th17 ↓		
	IL-17A ↓		
	DAS28-ESR ↓		
	VAS ↓		
Ergocalciferol	Treg ↑	NCT04472481	[100]
	DAS28-CRP↓		

Abbreviations: hrIL-2 – human recombinant Interleukin 2; MTX – Methotrexate; BM-MSCs – Bone Marrow-derived Mesencymal Stem Cells; CGI - Clinical Global Impression; VAS – Visual Analogue Scale; CRP – C-reactive Protein; ACR – American College of Rheumatology 20 Response Criteria; CDAI - Clinical Disease Activity Index; SDAI - Simplified Disease Activity Index; DAS28 - Disease Activity Score; ESR - Erythrocyte Sedimentation Rate.

* Official title of NCT01988506 trial is „Induction of Regulatory T Cells by Low Dose IL-2 in Autoimmune and Inflammatory Diseases (TRANSREG)”. The aim of TRANSREG study was to compare biological and clinical responses to the administration of low doses IL-2 across 14 selected pathologies: Rheumatoid Arthritis, Ankylosing Spondylitis, Systemic Lupus Erythematosus, Psoriasis, Behcet's Disease, Granulomatosis with Polyangiitis, Takayasu's Disease, Crohn's Disease, Ulcerative Colitis, Autoimmune Hepatitis, Sclerosing Cholangitis, Sjögren Syndrome, Systemic Sclerosis and Idiopathic Thrombocytopenic Purpura.

Second strategy involves ACT, based mostly on polyclonal autologous lymphocytes (aside from GVHD patients – in this group allogeneic (donor) regulatory T cells are isolated and administered to the patient - recipient). This protocol has been efficiently implemented in number of clinical trials: completed (kidney transplant, T1DM) or ongoing (GVHD, SLE) [7,48,[101], [102], [103]]. There has been no officially registered clinical trial involving adoptive Tregs transfer in RA patients till the end of 2023. Interestingly, this situtation changed in March 2024, when Sonoma Biotherapeutics Inc. announced the recruitment of participants to Phase 1 Trial *“Study of Single Doses of SBT777101 in Subjects With Rheumatoid Arthritis”*. SBT777101 is an experimental biological treatment that consist of autologous CAR Treg lymphocytes [92].

## 6 Allogeneic Tregs ACT as a potential treatment method for RA

The great success of ACT in many autoimmune conditions (with T1DM being the leader in the field) made rheumatoid arthritis destined to become the next target. RA causes the impairment of all aspects of QOL (Quality of life) and inevitably leads to mental health, social, environmental and sexual dysfunctions [104]. With its extreme negative impact on society, it is only a matter of time before we witness widespread application of Treg-based ACT in clinical trials. It is crucial to carefully choosea suitable study design and methodology, reflectng the aims of the research, and reach only for the protocols based on the latest scientific findings, to provide the most transparent and critical evidence base for evaluating the safety and efficacy of new medical product. We are no longer grasping in the dark for answers in the mysterious world of autoimmune regulation. The last couple of years were extremely productive for researchers specialized in the therapeutic potential of Tregs. It seems unwise to copy some fundamentally false assumptions that lead to vague and questionable outcomes. With our current knowledge, we should re-consider the use of autologous Tregs ACT in the first in-man studies.

It has been repeatedly proven that Tregs in RA patients present different kinds and levels of intrinsic dysfunctions [105]. In these settings, expanding and adoptively transferring defective regulatory cells seems to be of limited benefit, unless we develop the way to stabilize their phenotype [106,107]. It is questionable how objectively valuable is the real potential of these cells, since we deal with only the defective ones. Taking into consideration that implementation of healthy donor allogeneic Tregs has been successful in the trials involving autoimmune and hematologic conditions, we suggest to incorporate the same protocol in RA research [Table 3].

**Table 3:** Clinical trials involving allogeneic Tregs ACT as a treatment method of different diseases. Our investigation was conducted at the beginning of June 2024, using data from ClinicalTrials.gov registry, with „allogeneic Tregs” as the applied search criterion. The result showed 22 clinical trials; 8 of the studies turned out to be irrelevant to the topic of allogeneic Tregs ACT. Most of the remaining trials involved adoptive transfer of donor Tregs in the treatment of GVHD. Interestingly, 3 out of 5 actively recruiting studies are not focused on hematologic conditions.

**ClinicalTrials.gov ID**	**Intervention/Treatment**	**Medical condition**	**Study Start – Study Completion**	**Study Type/Phase**	**Enrollment**	Status
NCT01795573	donor Tregs	GVHD	2014-10-29- 2020-08-14	Interventional Phase 1	38	completed
NCT06052436	allogeneic thyTregs	SIRS ARDS COVID-19	2023-06-27-2027-12-31 (estimated)	Interventional Phase 1 Phase 2	24 (estimated)	recruiting
NCT01937468	donor Tregs + IL-2	GVHD	2013-11-2024-12 (estimated)	Interventional Phase 1	25	active, not recruiting
NCT03977103	High dose irradiation conditioning + donor Tregs/Tcon + HSCT	Hematologic Malignancies	2014-02-2023-02-28	Interventional Phase 2	80	unknown status
NCT01903473	donor Tregs + IL-2 + rapamycin	GVHD	2013-07-2022-09-12	Interventional Phase 2	19	terminated (slow recruitment)
NCT02749084	donor Tregs	GVHD	2016-08- 2022-06	Interventional Phase 1 Phase 2	18	completed
NCT05993611	Allogeneic CAR-Tregs	GVHD Hematologic Malignancies	2024-06-17-2028-05-21 (estimated)	Interventional Phase 1	27 (estimated)	recruiting
NCT01660607	donor Tregs + Tcon	Hematologic Malignancies	2011-12-2023-12-20	Interventional Phase 1 Phase 2	68	completed
NCT04482699	allogeneic hybrid Treg/Th2 cell (RAPA-501-ALLO)	COVID-19-related ARDS	2020-12-30-2021-09-13	Interventional Phase 1 Phase 2	1	terminated (change in eligible patient population)
NCT05423691	allogeneic Tregs (CK0804)	Myelofibrosis	2022-12-27-2024-04-30 (estimated)	Interventional Phase 1	24 (estimated)	recruiting
NCT02385019	donor Tregs	GVHD	2015-03-2019-12	Interventional Phase 1 Phase 2	22	unknown status
NCT05695521	UCB-derived allogeneic Tregs (CK0803)	ALS	2023-04-03-2027-12 (estimated)	Interventional Phase 1	66 (estimated)	recruiting
NCT05088356	donor Tregs + HSCT + Tcon	GVHD Hematologic Malignancies	2021-09-07-2025-12 (estimated)	Interventional Phase 1	40 (estimated)	recruiting
NCT02423915	fucosylated UCB-derived Tregs	GVHD	2015-07-30-2020-10-06	Interventional Phase 1	5	completed

Abbreviations: thyTregs – thymic regulatory T lymphocytes; Tcon – conventional T lymphocytes; HSCT - Hematopoietic Stem Cell Transplantation; GVHD – Graft versus Host Disease; SIRS – Systemic Inflammatory Response Syndrome; ARDS – Acute Respiratory Distress Syndrome; ALS - Amyotrophic Lateral Sclerosis.

Interestingly, allogeneic cells has already been used in the innovative therapies of RA – mesenchymal stem cells (MSCs) obtained from adipose tissue or umbilical cord have become one of the most promising approaches [108,109]. MSCs act as the immunosuppressive agents and, because of their important immunoprivileged properties, remain safe for allogeneic use. This results from MSCs having extremely low levels of major histocompatibility complex (MHC) class I and II molecules expression [110]. Somehow similar approach has been developed in our group where autologous Tregs were expanded in the presence of allogeneic mismatched MSC, which improved significantly the regulatory potential of Tregs [111]. Compared to mesenchymal, allogeneic Tregs ACT represent a challenge since administration of unmodified donor cells, unmatched in HLA complex, are likely to trigger sensitization or acute rejection by recipient immune system [83].

The obstacle of HLA mismatch could be overcomed by application of allogeneic Tregs derived from umbilical cord blood (UCB). The successful use of partially HLA-mismatched unrelated donor UCB as the source of hematopoietic stem cells has been reported as early as 1996 [112]. After the decades of studies, unrelated donor UCB has become the commonly acceptable alternative to HLA-matched bone marrow in the transplants among patients suffering from malignancies, BM failure, immunodeficiencies, etc [113]. Since UCB is easier to match than any other source of potentially therapeutic allogeneic cells, it has been widely and willingly used by researchers, providing some interesting findings, even concerning the recent pandemic [114]. Some newest reports show that the administration of allogeneic cord blood Tregs could be successfully used in the treatment of COVID-19 ARDS (Acute Respiratory Distress Syndrome) [115]. UCB is particularly alluring for clinical trials because it is the non-controversial and absolutely ethical source of stem cells collected post-birth, which prevents any political or religious issues. Obviously, cord blood has some limitations as the source of regulatory T lymphocytes, if they were to be used as a commonly accepted option for RA treatment. Cord-blood banking system is still growing, with The World Marrow Donor Association incharge, but faces many obstacles, such as financial issues or limited implementation of UCB banking in some countries [116].

## **7** Conclusions

Though still largely an experimental procedure, Treg-based adoptive cell therapy has recently become a clinical reality. Results from in-human trials are encouraging, but many questions remain to be addressed before this approach becomes routinely applicable to RA patients. We believe that the key to achieving satisfactory results of ACT in RA is implementation of the lymphocytes of allogeneic origin, not restricted by pathologic changes triggred by ongoing inflammatory processes.

The ideal situation in the distant future would be setting up a GMP(Good Manufacturing Practice)-compatible allogeneic cell banking system to cover large populations of patients – similar to hematopoietic stem cell transplantation (HSCT) strategies, exploited by physicians for decades [117]. The extensive use of immune cells from unrelated donors, optimally matched in MHC haplotypes, would offer many advantages over autologous cells, including improved cost-effectiveness, broader availability and higher quality of the product. Furthermore, allogeneic approach has the potential to provide a ready to use “off the shelf” immunotherapeutic, such that a single manufacturing run would allow multiple administration of the product. Likewise, by increasing the scale of production and creating a specific cell banking system, average cost per patient would decrease while the availability of therapeutic agent would increase [10,118]. The alternative for this ‘natural source’ of allogeneic Tregs might become Tregs with genetically engineered HLA that we are currently developing in our group. The idea is to create universal Treg donor lines by switching off HLA genes with either viral vectors or CRISPR technology [9,119].

Within the past decade, we have witnessed the transformative therapeutic potential of adoptive therapy for autoimmune conditions. However, at this point, we are still very far from clinical utilization of Tregs ACT on a bigger scale. At first we should focus on smaller trials, with carefully planned study design and methodology, to shed light on the exact mechanisms responsible for lymphocyte regulation in RA. This data would inevitably provide some further evidence of Tregs efficiency and safety in clinical practice. As long as we reach for defective autologous Tregs, proven abnormal or even pro-inflammatory, our results will not bring answers, only more pressing questions.

## Data availability statement

Data included in this article and all supporting materials are referenced in article.

Data regarding clinical trials analyzed in this article are publicly available in ClinicalTrials.gov database.

## Consent statement/Ethical approval

Not applicable.

## Funding

This review received no specific grant from any funding agency in the public, commercial, or not-for-profit sectors.

## CRediT authorship contribution statement

**Joanna Chmiel:** Writing – original draft, Investigation, Conceptualization. **Mariusz Stasiak:** Writing – review & editing, Investigation. **Maria Skrzypkowska:** Validation, Investigation. **Lucjan Samson:** Supervision, Resources. **Piotr Łuczkiewicz:** Supervision. **Piotr Trzonkowski:** Writing – review & editing, Project administration.

## Declaration of competing interest

The authors declare that they have no known competing financial interests or personal relationships that could have appeared to influence the work reported in this paper. The work described in this article has not been published previously, it is not under consideration for publication elsewhere, its publication is approved by all the authors and tacitly or explicitly by the responsible authorities where the work was carried out, and if accepted, it will not be published elsewhere in the same form, in English or in any other language, including electronically without the written consent of the copyright-holder.

## References

[1] Theis K.A., Roblin D.W., Helmick C.G., Luo R. (2018 Jan). Prevalence and causes of work disability among working-age U.S. adults, 2011-2013, NHIS. Disabil Health J.

[2] Sokka T. (2010). Work disability remains a major problem in rheumatoid arthritis in the 2000s: data from 32 countries in the QUEST-RA study. Arthritis Res. Ther..

[3] Smolen (2020 Jun). EULAR recommendations for the management of rheumatoid arthritis with synthetic and biological disease-modifying antirheumatic drugs: 2019 update. Ann. Rheum. Dis..

[4] Lewis M.J. (2019 Aug 27). Molecular portraits of early rheumatoid arthritis identify clinical and treatment response phenotypes. Cell Rep..

[5] Liu S., Ma H., Zhang H., Deng C., Xin P. (2021 Sep). Recent advances on signaling pathways and their inhibitors in rheumatoid arthritis. Clin Immunol.

[6] Koga T., Kawakami A., Tsokos G.C. (2021 Apr). Current insights and future prospects for the pathogenesis and treatment for rheumatoid arthritis. Clin Immunol.

[7] Goswami T.K. (2022 Dec 31). Regulatory T cells (Tregs) and their therapeutic potential against autoimmune disorders - advances and challenges. Hum Vaccin Immunother.

[8] Okeke E.B., Uzonna J.E. (2019 Apr 9). The pivotal role of regulatory T cells in the regulation of innate immune cells. Front. Immunol..

[9] Raffin C., Vo L.T., Bluestone J.A. (2020 Mar). Treg cell-based therapies: challenges and perspectives. Nat. Rev. Immunol..

[10] Selck C., Dominguez-Villar M. (2021 May 14). Antigen-specific regulatory T cell therapy in autoimmune diseases and transplantation. Front. Immunol..

[11] Eberlein T.J. (August 1982). Regression of a disseminated syngeneic solid tumor by systemic transfer of lymphoid cells expanded in interleukin 2. J. Exp. Med..

[12] Bi Y., Kong R., Peng Y., Yu H., Zhou Z. (2023 Oct). Umbilical cord blood and peripheral blood-derived regulatory T cells therapy: progress in type 1 diabetes. Clin Immunol.

[13] Esparcia-Pinedo L., Lancho-Sánchez Á., Tsukalov I., Pacheco M.I., Martínez-Fleta P., Pérez-Miés B., Palacios-Calvo J., Sánchez-Madrid F., Martín-Gayo E., Alfranca A. (2023 Nov). T regulatory lymphocytes specific for SARS-CoV-2 display increased functional plasticity. Clin Immunol.

[14] Shevyrev D., Tereshchenko V. (2020 Jan 14). Treg heterogeneity, function, and homeostasis. Front. Immunol..

[15] Rajendeeran A., Tenbrock K. (2021 Oct 30). Regulatory T cell function in autoimmune disease. J Transl Autoimmun.

[16] Göschl L., Scheinecker C., Bonelli M. (2019 May). Treg cells in autoimmunity: from identification to Treg-based therapies. Semin. Immunopathol..

[17] Arellano B., Graber D.J., Sentman C.L. (2016 Aug). Regulatory T cell-based therapies for autoimmunity. Discov. Med..

[18] Sakaguchi S., Sakaguchi N., Asano M., Itoh M., Toda M. (1995 Aug 1). Immunologic self-tolerance maintained by activated T cells expressing IL-2 receptor alpha-chains (CD25). Breakdown of a single mechanism of self-tolerance causes various autoimmune diseases. J. Immunol..

[19] Miyara M. (2009 Jun 19). Functional delineation and differentiation dynamics of human CD4+ T cells expressing the FoxP3 transcription factor. Immunity.

[20] Shevach E.M., Thornton A.M. (2014 May). tTregs, pTregs, and iTregs: similarities and differences. Immunol. Rev..

[21] Chekol Abebe E., Asmamaw Dejenie T., Mengie Ayele T., Dagnew Baye N., Agegnehu Teshome A., Tilahun Muche Z. (2021 Jan 12). The role of regulatory B cells in health and diseases: a systemic review. J. Inflamm. Res..

[22] Liston A., Aloulou M. (2022 Jul). A fresh look at a neglected regulatory lineage: CD8^+^Foxp3^+^ Regulatory T cells. Immunol. Lett..

[23] Moro-García M.A., Mayo J.C., Sainz R.M., Alonso-Arias R. (2018 Mar 1). Influence of inflammation in the process of T lymphocyte differentiation: proliferative, metabolic, and oxidative changes. Front. Immunol..

[24] Komatsu N., Okamoto K., Sawa S., Nakashima T., Oh-hora M., Kodama T., Tanaka S., Bluestone J.A., Takayanagi H. (2014 Jan). Pathogenic conversion of Foxp3+ T cells into TH17 cells in autoimmune arthritis. Nat Med.

[25] Yang L., Wang G., Xia H. (2020 Oct). Molecular mechanism for impaired suppressive function of Tregs in autoimmune diseases: a summary of cell-intrinsic and cell-extrinsic factors. J. Cell Mol. Med..

[26] Guo H., Xun L., Zhang R., Hu F., Luan J., Lao K., Wang X., Gou X. (2019 Oct). Stability and inhibitory function of Treg cells under inflammatory conditions in vitro. Exp. Ther. Med..

[27] Zhang J., Liu H., Chen Y., Liu H., Zhang S., Yin G., Xie Q. (2024 Jan 23). Augmenting regulatory T cells: new therapeutic strategy for rheumatoid arthritis. Front. Immunol..

[28] Tortola L., Pawelski H., Sonar S.S., Ampenberger F., Kurrer M., Kopf M. (2019 Jun). IL-21 promotes allergic airway inflammation by driving apoptosis of FoxP3^+^ regulatory T cells. J. Allergy Clin. Immunol..

[29] Nie H., Zheng Y., Li R., Guo T.B., He D., Fang L., Liu X., Xiao L., Chen X., Wan B., Chin Y.E., Zhang J.Z. (2013 Mar). Phosphorylation of FOXP3 controls regulatory T cell function and is inhibited by TNF-α in rheumatoid arthritis. Nat Med.

[30] Balendran T., Lim K., Hamilton J.A., Achuthan A.A. (2023 Jun 29). Targeting transcription factors for therapeutic benefit in rheumatoid arthritis. Front. Immunol..

[31] Simon L.S., Taylor P.C., Choy E.H., Sebba A., Quebe A., Knopp K.L., Porreca F. (2021 Feb). The Jak/STAT pathway: a focus on pain in rheumatoid arthritis. Semin. Arthritis Rheum..

[32] Duan T., Du Y., Xing C., Wang H.Y., Wang R.F. (2022 Mar 3). Toll-like receptor signaling and its role in cell-mediated immunity. Front. Immunol..

[33] Tomaszewicz M., Ronowska A., Zieliński M., Jankowska-Kulawy A., Trzonkowski P. (2023 Mar 3). T regulatory cells metabolism: the influence on functional properties and treatment potential. Front. Immunol..

[34] Jang S.W., Hwang S.S., Kim H.S., Kim M.K., Lee W.H., Hwang S.U., Gwak J., Yew S.K., Flavell R.A., Lee G.R. (2019 Dec 17). Homeobox protein Hhex negatively regulates Treg cells by inhibiting Foxp3 expression and function. Proc Natl Acad Sci U S A.

[35] Xu Y., Melo-Cardenas J., Zhang Y., Gau I., Wei J., Montauti E., Zhang Y., Gao B., Jin H., Sun Z., Lee S.M., Fang D. (2019 Mar 7). The E3 ligase Hrd1 stabilizes Tregs by antagonizing inflammatory cytokine-induced ER stress response. JCI Insight.

[36] Dong Y., Yang C., Pan F. (2021 Apr 12). Post-Translational regulations of Foxp3 in Treg cells and their therapeutic applications. Front. Immunol..

[37] Le Menn G., Jabłońska A., Chen Z. (2022 Jun). The effects of post-translational modifications on Th17/Treg cell differentiation. Biochim. Biophys. Acta Mol. Cell Res..

[38] Peng X., Wang Q., Li W., Ge G., Peng J., Xu Y., Yang H., Bai J., Geng D. (2023 Jan 24). Comprehensive overview of microRNA function in rheumatoid arthritis. Bone Res.

[39] Liu C., Li N., Liu G. (2020 Apr 2). The role of MicroRNAs in regulatory T cells. J Immunol Res.

[40] Zhang Y., Yang M., Xie H., Hong F., Yang S. (2023 Jun 30). Role of miRNAs in rheumatoid arthritis therapy. Cells.

[41] Peng Q., Ratnasothy K., Boardman D.A., Jacob J., Tung S.L., McCluskey D., Smyth L.A., Lechler R.I., Dorling A., Lombardi G. (2019 Jun 18). Protease activated receptor 4 as a novel modulator of regulatory T cell function. Front. Immunol..

[42] Du X., Shi H., Li J., Dong Y., Liang J., Ye J., Kong S., Zhang S., Zhong T., Yuan Z., Xu T., Zhuang Y., Zheng B., Geng J.G., Tao W. (2014 Feb 15). Mst1/Mst2 regulate development and function of regulatory T cells through modulation of Foxo1/Foxo3 stability in autoimmune disease. J. Immunol..

[43] Shen C.R., Yang W.C., Chen H.W. (2014 Jan). The fate of regulatory T cells: survival or apoptosis. Cell. Mol. Immunol..

[44] Hu W., Wang Z.M., Feng Y. (2021). Regulatory T cells function in established systemic inflammation and reverse fatal autoimmunity. Nat. Immunol..

[45] Cvetanovich G.L., Hafler D.A. (2010 Dec). Human regulatory T cells in autoimmune diseases. Curr. Opin. Immunol..

[46] Conrad N. (2023 Jun 3). Incidence, prevalence, and co-occurrence of autoimmune disorders over time and by age, sex, and socioeconomic status: a population-based cohort study of 22 million individuals in the UK. Lancet.

[47] Finckh A. (2022 Oct). Global epidemiology of rheumatoid arthritis. Nat. Rev. Rheumatol..

[48] Yan S., Kotschenreuther K., Deng S., Kofler D.M. (2022 Sep 29). Regulatory T cells in rheumatoid arthritis: functions, development, regulation, and therapeutic potential. Cell. Mol. Life Sci..

[49] van Amelsfort J.M., Jacobs K.M., Bijlsma J.W., Lafeber F.P., Taams L.S. (2004 Sep). CD4(+)CD25(+) regulatory T cells in rheumatoid arthritis: differences in the presence, phenotype, and function between peripheral blood and synovial fluid. Arthritis Rheum..

[50] Morita T., Shima Y., Wing J.B., Sakaguchi S., Ogata A., Kumanogoh A. (2016 Sep 13). The proportion of regulatory T cells in patients with rheumatoid arthritis: a meta-analysis. PLoS One.

[51] Valencia X., Stephens G., Goldbach-Mansky R., Wilson M., Shevach E.M., Lipsky P.E. (2006 Jul 1). TNF downmodulates the function of human CD4+CD25hi T-regulatory cells. Blood.

[52] Nie H., Zheng Y., Li R., Guo T.B., He D., Fang L., Liu X., Xiao L., Chen X., Wan B., Chin Y.E., Zhang J.Z. (2013 Mar). Phosphorylation of FOXP3 controls regulatory T cell function and is inhibited by TNF-α in rheumatoid arthritis. Nat Med.

[53] Skrzypkowska M. (2022 Apr). Cytokines and chemokines multiplex analysis in patients with low disease activity rheumatoid arthritis. Rheumatol. Int..

[54] Jiang Q., Yang G., Liu Q., Wang S., Cui D. (2021 Apr 1). Function and role of regulatory T cells in rheumatoid arthritis. Front. Immunol..

[55] van Amelsfort J.M., van Roon J.A., Noordegraaf M., Jacobs K.M., Bijlsma J.W., Lafeber F.P., Taams L.S. (2007 Mar). Proinflammatory mediator-induced reversal of CD4+,CD25+ regulatory T cell-mediated suppression in rheumatoid arthritis. Arthritis Rheum..

[56] Chakraborty S., Gupta R., Kubatzky K.F., Kar S., Kraus F.V., Souto-Carneiro M.M., Lorenz H.M., Kumar P., Kumar V., Mitra D.K. (2023 Dec). Negative impact of Interleukin-9 on synovial regulatory T cells in rheumatoid arthritis. Clin Immunol.

[57] Corthay A. (2009 Oct). How do regulatory T cells work?. Scand. J. Immunol..

[58] Li Y.S., Luo W., Zhu S.A., Lei G.H. (2017 Mar 30). T cells in osteoarthritis: alterations and beyond. Front. Immunol..

[59] Moradi B. (2014 Apr 17). CD4⁺CD25⁺/highCD127low/⁻ regulatory T cells are enriched in rheumatoid arthritis and osteoarthritis joints--analysis of frequency and phenotype in synovial membrane, synovial fluid and peripheral blood. Arthritis Res. Ther..

[60] Xia N. (2020 Nov 17). A unique population of regulatory T cells in heart potentiates cardiac protection from myocardial infarction. Circulation.

[61] Wang H.Y., Ye J.R., Cui L.Y., Chu S.F., Chen N.H. (2022 Jan). Regulatory T cells in ischemic stroke. Acta Pharmacol. Sin..

[62] Shi L. (2021 Jul 13). Treg cell-derived osteopontin promotes microglia-mediated white matter repair after ischemic stroke. Immunity.

[63] Das S., Rai G., Sood V., Singh P.K., Tyagi A., Salhotra R., Gupta C., Jaggi V.K., Dar S.A., Ansari M.A. (2023 Sep 30). Incompetent memory immune response in severe COVID-19 patients under treatment. Heliyon.

[64] Togashi Y., Shitara K., Nishikawa H. (2019 Jun). Regulatory T cells in cancer immunosuppression - implications for anticancer therapy. Nat. Rev. Clin. Oncol..

[65] Verma A., Mathur R., Farooque A., Kaul V., Gupta S., Dwarakanath B.S. (2019 Dec 24). T-regulatory cells in tumor progression and therapy. Cancer Manag. Res..

[66] Dwivedi M., Tiwari S., Kemp E.H., Begum R. (2022 Aug 27). Implications of regulatory T cells in anti-cancer immunity: from pathogenesis to therapeutics. Heliyon.

[67] Lykhopiy V., Malviya V., Humblet-Baron S. (2023). IL-2 immunotherapy for targeting regulatory T cells in autoimmunity. Genes Immun.

[68] Gliwiński M., Iwaszkiewicz-Grześ D., Trzonkowski P. (2017 Aug). Cell-based therapies with T regulatory cells. BioDrugs.

[69] Giganti G., Atif M., Mohseni Y., Mastronicola D., Grageda N., Povoleri G.A., Miyara M., Scottà C. (2021 Jan). Treg cell therapy: how cell heterogeneity can make the difference. Eur. J. Immunol..

[70] Gu J., Shao Q., Zhou J., Chen Q., Lu L. (2022 Dec 16). Protocol for in vitro isolation, induction, expansion, and determination of human natural regulatory T cells and induced regulatory T cells. STAR Protoc.

[71] Trzonkowski P. (2015 Sep 9). Hurdles in therapy with regulatory T cells. Sci. Transl. Med..

[72] Fuchs A. (2018 Jan 15). Minimum information about T regulatory cells: a step toward reproducibility and standardization. Front. Immunol..

[73] Abhishek K., Nidhi M., Chandran S., Shevkoplyas S.S., Mohan C. (2023 Jun). Manufacturing regulatory T cells for adoptive cell therapy in immune diseases: a critical appraisal. Clin Immunol.

[74] Reinhardt J., Sharma V., Stavridou A., Lindner A., Reinhardt S., Petzold A., Lesche M., Rost F., Bonifacio E., Eugster A. (2022 May). Distinguishing activated T regulatory cell and T conventional cells by single-cell technologies. Immunology.

[75] Balcerek J., Shy B.R., Putnam A.L., Masiello L.M., Lares A., Dekovic F., Acevedo L., Lee M.R., Nguyen V., Liu W., Paruthiyil S., Xu J., Leinbach A.S., Bluestone J.A., Tang Q., Esensten J.H. (2021 Nov 18). Polyclonal regulatory T cell manufacturing under cGMP: a decade of experience. Front. Immunol..

[76] Skartsis N., Peng Y., Ferreira L.M.R., Nguyen V., Ronin E., Muller Y.D., Vincenti F., Tang Q. (2021 Dec 23). IL-6 and TNFα drive extensive proliferation of human Tregs without compromising their lineage stability or function. Front. Immunol..

[77] Someya K., Nakatsukasa H., Ito M., Kondo T., Tateda K.I., Akanuma T., Koya I., Sanosaka T., Kohyama J., Tsukada Y.I., Takamura-Enya T., Yoshimura A. (2017 Aug 1). Improvement of Foxp3 stability through CNS2 demethylation by TET enzyme induction and activation. Int. Immunol..

[78] Thangavelu G., Andrejeva G., Bolivar-Wagers S. (2022). Retinoic acid signaling acts as a rheostat to balance Treg function. Cell. Mol. Immunol..

[79] Dawson N.A.J., Levings M.K. (2017 Sep). Antigen-specific regulatory T cells: are police CARs the answer?. Transl. Res..

[80] Brunstein C.G. (2011 Jan 20). Infusion of ex vivo expanded T regulatory cells in adults transplanted with umbilical cord blood: safety profile and detection kinetics. Blood.

[81] Wang C., Wang J., Che S., Zhao H. (2023 Nov 7). CAR-T cell therapy for hematological malignancies: history, status and promise. Heliyon.

[82] Amini L., Greig J., Schmueck-Henneresse M., Volk H.D., Bézie S., Reinke P., Guillonneau C., Wagner D.L., Anegon I. (2021 Feb 24). Super-treg: toward a new era of adoptive Treg therapy enabled by genetic modifications. Front. Immunol..

[83] McCallion O., Bilici M., Hester J., Issa F. (2023 Mar 16). Regulatory T-cell therapy approaches. Clin. Exp. Immunol..

[84] Sterner R.C., Sterner R.M. (2021 Apr 6). CAR-T cell therapy: current limitations and potential strategies. Blood Cancer J..

[85] Stucchi A., Maspes F., Montee-Rodrigues E., Fousteri G. (2024 Apr). Engineered Treg cells: the heir to the throne of immunotherapy. J. Autoimmun..

[86] Ajeganova S., Huizinga T. (2017 Oct). Sustained remission in rheumatoid arthritis: latest evidence and clinical considerations. Ther Adv Musculoskelet Dis.

[87] Gao Y., Gao Y.N., Wang M.J., Zhang Y., Zhang F.Q., He Z.X., Chen W., Li H.C., Xie Z.J., Wen C.P. (2023 Apr 28). Efficacy and safety of tofacitinib combined with methotrexate in the treatment of rheumatoid arthritis: a systematic review and meta-analysis. Heliyon.

[88] Zhu H. (2018 Mar). Remission assessment of rheumatoid arthritis in daily practice in China: a cross-sectional observational study. Clin. Rheumatol..

[89] Hartemann A. (2013 Dec). Low-dose interleukin 2 in patients with type 1 diabetes: a phase 1/2 randomised, double-blind, placebo-controlled trial. Lancet Diabetes Endocrinol..

[90] von Spee-Mayer C. (2016 Jul). Low-dose interleukin-2 selectively corrects regulatory T cell defects in patients with systemic lupus erythematosus. Ann. Rheum. Dis..

[91] Zheng X., Su R., Hu F., Liu Y., Li X., Gao C., Wang C. (2022 Nov). Low-dose IL-2 therapy restores imbalance between Th17 and regulatory T cells in patients with the dermatomyositis combined with EBV/CMV viremia. Autoimmun. Rev..

[92] ClinicalTrials.gov [Internet] (2000 Feb 29). NCT06201416.

[93] Rosenzwajg M., Lorenzon R., Cacoub P., Pham H.P., Pitoiset F., El Soufi K., Ribet C., Bernard C., Aractingi S., Banneville B., Beaugerie L., Berenbaum F., Champey J., Chazouilleres O., Corpechot C., Fautrel B., Mekinian A., Regnier E., Saadoun D., Salem J.E., Sellam J., Seksik P., Daguenel-Nguyen A., Doppler V., Mariau J., Vicaut E., Klatzmann D. (2019 Feb). Immunological and clinical effects of low-dose interleukin-2 across 11 autoimmune diseases in a single, open clinical trial. Ann. Rheum. Dis..

[94] Lorenzon R., Ribet C., Pitoiset F., Aractingi S., Banneville B., Beaugerie L., Berenbaum F., Cacoub P., Champey J., Chazouilleres O., Corpechot C., Fautrel B., Mekinian A., Regnier E., Saadoun D., Salem J.E., Sellam J., Seksik P., Vicaut E., Rosenzwajg M., Klatzmann D. (2024 Apr). The universal effects of low-dose interleukin-2 across 13 autoimmune diseases in a basket clinical trial. J. Autoimmun..

[95] Carvajal Alegria G., Cornec D., Saraux A., Devauchelle-Pensec V., Jamin C., Hillion S., Pers J.O., Pochard P. (2021 Jul 15). Abatacept promotes regulatory B cell functions, enhancing their ability to reduce the Th1 response in rheumatoid arthritis patients through the production of IL-10 and TGF-β. J. Immunol..

[96] Zhang X., Miao M., Zhang R. (2022). Efficacy and safety of low-dose interleukin-2 in combination with methotrexate in patients with active rheumatoid arthritis: a randomized, double-blind, placebo-controlled phase 2 trial. Sig Transduct Target Ther.

[97] Ghoryani M., Shariati-Sarabi Z., Tavakkol-Afshari J., Ghasemi A., Poursamimi J., Mohammadi M. (2019 Jan). Amelioration of clinical symptoms of patients with refractory rheumatoid arthritis following treatment with autologous bone marrow-derived mesenchymal stem cells: a successful clinical trial in Iran. Biomed. Pharmacother..

[98] Rahimi Khorashad M., Ghoryani M., Gowhari Shabgah A., Shariati-Sarabi Z., Tavakkol Afshari J., Mohammadi M. (2023 Apr 30). The effects of mesenchymal stem cells on the gene expression of TGF-beta and IFN-gamma in patients with rheumatoid arthritis. Iran. J. Allergy, Asthma Immunol..

[99] Alavi M., Tavakkol-Afshari J., Shariati-Sarabi Z., Shabgah A.G., Ghoryani M., Ghasemi A., Mohammadi M. (2020 Dec 30). Intravenous injection of autologous bone marrow-derived mesenchymal stem cells on the gene expression and plasma level of CCL5 in refractory rheumatoid arthritis. J. Res. Med. Sci..

[100] El-Banna H.S., Gado S.E. (2020 Aug). Vitamin D: does it help Tregs in active rheumatoid arthritis patients. Expert Rev Clin Immunol..

[101] Trzonkowski P., Bieniaszewska M., Juścińska J., Dobyszuk A., Krzystyniak A., Marek N., Myśliwska J., Hellmann A. (2009 Oct). First-in-man clinical results of the treatment of patients with graft versus host disease with human ex vivo expanded CD4+CD25+CD127- T regulatory cells. Clin Immunol.

[102] Marek-Trzonkowska N. (2012 Sep). Administration of CD4+CD25highCD127- regulatory T cells preserves β-cell function in type 1 diabetes in children. Diabetes Care.

[103] Chwojnicki K. (2021 Jan). Administration of CD4^+^CD25^high^CD127^-^FoxP3^+^ regulatory T cells for relapsing-remitting multiple sclerosis: a phase 1 study. BioDrugs.

[104] Martinec R., Pinjatela R., Balen D. (2019 Mar). Quality of life in patients with rheumatoid arthritis - a preliminary study. Acta Clin. Croat..

[105] Cribbs A.P., Kennedy A., Penn H., Read J.E., Amjadi P., Green P., Syed K., Manka S.W., Brennan F.M., Gregory B., Williams R.O. (2014 Sep). Treg cell function in rheumatoid arthritis is compromised by ctla-4 promoter methylation resulting in a failure to activate the indoleamine 2,3-dioxygenase pathway. Arthritis Rheumatol..

[106] Marek N., Bieniaszewska M., Krzystyniak A., Juścińska J., Myśliwska J., Witkowski P., Hellmann A., Trzonkowski P. (2011). The time is crucial for ex vivo expansion of T regulatory cells for therapy. Cell Transplant..

[107] Marek-Trzonkowska N., Piekarska K., Filipowicz N., Piotrowski A., Gucwa M., Vogt K., Sawitzki B., Siebert J., Trzonkowski P. (2017 Sep 20). Mild hypothermia provides Treg stability. Sci. Rep..

[108] Álvaro-Gracia J.M. (2017 Jan). Intravenous administration of expanded allogeneic adipose-derived mesenchymal stem cells in refractory rheumatoid arthritis (Cx611): results of a multicentre, dose escalation, randomised, single-blind, placebo-controlled phase Ib/IIa clinical trial. Ann. Rheum. Dis..

[109] Harna B. (2023 Feb 1). Mesenchymal stromal cell therapy for patients with rheumatoid arthritis. Exp. Cell Res..

[110] Lalu M.M., McIntyre L., Pugliese C., Fergusson D., Winston B.W., Marshall J.C., Granton J., Stewart D.J., Canadian Critical Care Trials Group (2012). Safety of cell therapy with mesenchymal stromal cells (SafeCell): a systematic review and meta-analysis of clinical trials. PLoS One.

[111] Piekarska K. (2022 Feb 14). Mesenchymal stem cells transfer mitochondria to allogeneic Tregs in an HLA-dependent manner improving their immunosuppressive activity. Nat. Commun..

[112] Kurtzberg J., Laughlin M., Graham M.L., Smith C., Olson J.F., Halperin E.C., Ciocci G., Carrier C., Stevens C.E., Rubinstein P. (1996 Jul 18). Placental blood as a source of hematopoietic stem cells for transplantation into unrelated recipients. N. Engl. J. Med..

[113] Barker J.N., Davies S.M., DeFor T., Ramsay N.K., Weisdorf D.J., Wagner J.E. (2001 May 15). Survival after transplantation of unrelated donor umbilical cord blood is comparable to that of human leukocyte antigen-matched unrelated donor bone marrow: results of a matched-pair analysis. Blood.

[114] Wang Q., Wang Y., Chang C., Ma F., Peng D., Yang S., An Y., Deng Q., Wang Q., Gao F., Wang F., Tang H., Qi X., Jiang X., Cai D., Zhou G. (2023 Jan 4). Comparative analysis of mesenchymal stem/stromal cells derived from human induced pluripotent stem cells and the cognate umbilical cord mesenchymal stem/stromal cells. Heliyon.

[115] Gladstone D.E. (2023 Jul 11). Randomized, double-blinded, placebo-controlled trial of allogeneic cord blood T-regulatory cells for treatment of COVID-19 ARDS. Blood Adv.

[116] Ballen K. (2017 May). Umbilical cord blood transplantation: challenges and future directions. Stem Cells Transl Med.

[117] Pakzad M. (2022 Oct). A roadmap for the production of a GMP-compatible cell bank of allogeneic bone marrow-derived clonal mesenchymal stromal cells for cell therapy applications. Stem Cell Rev Rep.

[118] Caldwell K.J., Gottschalk S., Talleur A.C. (2021 Jan 8). Allogeneic CAR cell therapy-more than a pipe dream. Front. Immunol..

[119] Johansen K.H. (2022 Jan). How CRISPR/Cas9 gene editing is revolutionizing T cell research. DNA Cell Biol..

